# Long-term immunity after HBV vaccine: shall we consider a change? A 20-year-follow-up study

**DOI:** 10.1093/eurpub/ckac129.052

**Published:** 2022-10-25

**Authors:** M Fonzo, I Amoruso, T Baldovin, A Trevisan, C Bertoncello

**Affiliations:** Hygiene and Public Health Unit, DCTVSP, University, Padua, Italy

## Abstract

**Introduction:**

Although vaccines against HBV have been available since the 1980s, the long-term immunity is still debated. When assessing immune persistence, a number of clearly defined variables must be taken into account. Often the expression ‘infant vaccination’ means the administration within the first year of life at any age, but a difference of a few months may imply a different antibody persistence over the years. This study assessed the anti-HBs titre 20 years after the primary vaccination course and estimated the effect of age at 1st dose and time interval between doses on long-term protection.

**Methods:**

Data on age, sex and date of administration were collected. Inclusion criteria: born to negative mother, 3-dose schedule, no previous HBV infection, age at enrolment 18-24 years; age at 1st dose 2-12 months. Titres ≥10IU/l were considered protective. A logistic regression was performed, adjusting for sex, follow-up time and date of 1st dose and analysis.

**Results:**

We included 5,485 participants (64% female). The mean anti-HBsAg increased from 46, 52, 85 to 193IU/l when the 1st dose was administered in the I, II, III or IV trimester of life, respectively. Similarly, the proportion of individuals with titre <10IU/l decreased from 51 to 18% between the two extreme quarters. The risk of a titre <10IU/l decreased with age at the 1st dose (AOR: 0.84; 95%CI: 0.78-0.91 per one-month increase) and time between the 2nd and 3rd doses (AOR: 0.89; 95%CI:0.85-0.94).

**Conclusions:**

The mere presence of a titre <10IU/l does not equate lack of protection. However, antibody levels are very different depending on the actual age of vaccination. One-month delay within the first year is associated with a -18% chance of a titre <10IU/l 20 years later. Although this information needs to be combined with local epidemiology and surveillance to obtain an informed risk-benefit balance, the implications from a public health and economic perspective may be diverse and worth considering.

**Key messages:**

• Still within the first year of life, a delay in the administration of the 1st dose of HBV vaccine and a longer time between the 2nd and 3rd dose imply a higher antibody persistence even 20 years later.

• Considering the local circulation of HBV and surveillance, this result could be taken into account to obtain an informed risk-benefit balance.

